# Analyzing Gait Dynamics and Recovery Trajectory in Lower Extremity Fractures Using Linear Mixed Models and Gait Analysis Variables

**DOI:** 10.3390/bioengineering12010067

**Published:** 2025-01-14

**Authors:** Mostafa Rezapour, Rachel B. Seymour, Suman Medda, Stephen H. Sims, Madhav A. Karunakar, Nahir Habet, Metin Nafi Gurcan

**Affiliations:** 1 Center for Artificial Intelligence Research, Wake Forest University School of Medicine, Winston-Salem, NC 27101, USA; 2 Department of Orthopaedic Surgery, Atrium Health Musculoskeletal Institute, Wake Forest University School of Medicine, Charlotte, NC 28210, USA; rachel.seymour@atriumhealth.org (R.B.S.); suman.medda1@atriumhealth.org (S.M.); stephen.sims@atriumhealth.org (S.H.S.); madhav.karunakar@atriumhealth.org (M.A.K.);

**Keywords:** gait analysis, recovery trajectory, lower extremity fractures, linear mixed models (LMM)

## Abstract

In a prospective study, we examined the recovery trajectory of patients with lower extremity fractures to better understand the healing process in the absence of complications. Using a chest-mounted inertial measurement unit (IMU) device for gait analysis and collecting patient-reported outcome measures, we focused on 12 key gait variables, including Mean Leg Lift Acceleration, Stance Time, and Body Orientation. We employed a linear mixed model (LMM) to analyze these variables over time, incorporating both fixed and random effects to account for individual differences and the time since injury. This model also adjusted for varying intervals between assessments. Our study provided insights into gait recovery across different fracture types using data from 318 patients who experienced no complications or readmissions during their recovery. Through LMM analysis, we found that Tibia-Distal fractures demonstrated the fastest recovery, particularly in terms of mobility and strength. Tibia-Proximal fractures showed balanced improvements in both mobility and stability, suggesting that rehabilitation should target both strength and balance. Femur fractures exhibited varied recovery, with Diaphyseal fractures showing clear improvements in stability, while Distal fractures reflected gains in limb strength but with some variability in stability. To examine patients with readmissions, we conducted a Chi-squared test of independence to determine whether there was a relationship between fracture type and readmission rates, revealing a significant association (*p* < 0.001). Pelvis fractures had the highest readmission rates, while Tibia-Diaphyseal and Tibia-Distal fractures were more prone to infections, highlighting the need for enhanced infection control strategies. Femur fractures showed moderate readmission and infection rates, indicating a mixed risk profile. In conclusion, our findings emphasize the importance of fracture-specific rehabilitation strategies, focusing on infection prevention and individualized treatment plans to optimize recovery outcomes.

## 1. Introduction

Orthopedic trauma to the lower extremities is prevalent in both military and civilian populations, with the main treatment goal being the restoration of previous function [1]. Rehabilitation involves early movement and weight-bearing, tailored by the type and fixation of the injury, though these can sometimes risk damaging the healing structures [2]. Protocols vary; for instance, calcaneal fractures generally require non-weight-bearing (NWB) for 6–12 weeks post-open reduction internal fixation (ORIF), whereas external fixation allows immediate weight-bearing [3,4]. Similarly, tibial plafond fractures usually involve a non-weight-bearing period of 6–12 weeks, with external fixation allowing for immediate weight-bearing [5].

Tibial shaft fractures have different options, with immediate weight-bearing permitted in both external and intramedullary fixation, but ORIF may require an NWB period of 6–12 weeks for comminuted or high-grade tibia fractures [6,7,8]. Similar considerations apply to femoral and pelvic fractures [9,10,11,12,13,14].

In addition to weight-bearing protocols, gait analysis plays a key role in assessing patient improvement and functionality following surgical treatment for lower extremity fractures [15]. Gait analysis is increasingly recognized and important in assessing rehabilitation progress and mobility for patients with lower extremity fractures [16]. The use of retro-reflective markers and high-speed cameras in gait analysis systems has significantly improved their accuracy and reliability [17,18].

While measurement technology has matured, computer models used for interpreting marker data are still evolving, with the Conventional Gait Model being commonly employed [1]. Floor-based photocell systems have been traditional methods for gait evaluation but may be impractical due to limitations. Wearable inertial measurement units (IMUs) offer an alternative, providing reliable gait parameters for effective rehabilitation tracking [19,20,21].

Warschawski et al. [19] found that patients with tibial plateau fractures exhibited persistent gait abnormalities such as slower walking speeds, reduced cadence, and shorter step lengths compared to healthy controls. Warschawski et al. [19] emphasized the effectiveness of computerized IMU-based gait analysis in providing precise data for evaluating long-term rehabilitation outcomes. Millar et al. [20] found that after surgery for tibial plateau fractures, patients experienced increased joint loads at the hip and ankle within the first six months, while knee joint loading showed significant improvement only during this period and then plateaued. Millar et al. [20] also demonstrated the effectiveness of gait analysis and biomechanical tools in accurately tracking recovery dynamics and identifying key milestones after tibial plateau fracture surgeries.

In our recent study [21], we demonstrated the potential of a wearable IMU to provide accurate, clinically relevant gait data for patients with lower extremity trauma in an outpatient clinic setting. The IMU data, when compared to the gold-standard Vicon video motion capture system, showed strong correlations across various gait parameters such as vertical acceleration, vertical displacement, and angular velocities (pitch and roll). The system was able to reliably measure and differentiate between normal and pathological gait patterns in trauma patients, capturing subtle asymmetries and compensatory movements that may not be easily detected through visual inspection alone. By providing quantitative data on gait parameters like stance time and vertical displacement, the IMU could serve as an efficient performance-based measure (PBM) to objectively assess gait outcomes and track patient recovery.

In another study, we pioneered the integration of supervised machine learning models with gait analysis to predict post-injury complications such as infection, malunion, and hardware irritation in individuals with lower extremity fractures [22]. We collected data using the IMU and developed a novel machine-learning algorithm named GUARD-XGBoost, which significantly outperformed other models. With an area under the curve (AUC) of 0.90 and accuracy rates of 86%, our study underscored the transformative potential of machine learning in orthopedic care.

In the current study, we aim to quantitatively assess the recovery trajectories of patients with lower extremity fractures by employing gait analysis to measure their mobility and functionality over time. Our primary objective is to leverage the chest-mounted IMU [21] to collect detailed gait data from patients who have sustained various types of fractures. The gait data collected with the IMU are analyzed using Linear Mixed Models (LMM) [23] to account for both fixed and random effects, thereby accommodating individual differences in baseline gait characteristics and the rate of recovery. This methodological choice enables us to discern the average effects of time since injury on specific gait variables, providing a robust statistical framework to assess and predict recovery patterns across diverse patient groups. Additionally, we employ the Chi-squared test [24] to investigate the association between fracture types and the frequency of readmissions, a key indicator of post-treatment complications.

## 2. Materials and Methods

Our study received approval from three successive Institutional Review Boards (IRBs): It was initially approved by the Carolinas HealthCare System IRB (IRB File #03-15-11E) on 18 March 2015, subsequently by the Atrium Health IRB (IRB File #03-15-11E), and is currently approved by the Wake Forest University School of Medicine IRB (IRB00082570). All methods were carried out in accordance with relevant guidelines and regulations. We obtained written informed consent from all participants or their legal guardian(s) prior to their involvement in any study-related activities.

Following approval from the institutional review board, we began identifying potential participants at an academic tertiary medical center. These were patients with lower extremity fractures located in the orthopedic trauma clinic after receiving treatment for their injuries at an urban Level 1 trauma center between 12/14/2015 and 09/12/2019. The enrolment process commenced before any performance tests were conducted. As part of the standard care, these patients had routine visits to the treating surgeon, during which we invited them to participate in our study. The participant demographics represented a broad range of ages, from 19 to 76 years, with a gender distribution of 65% male and 35% female.

The ethnicity mix included individuals from a variety of backgrounds, with 2% identifying as Hispanic, 90% non-Hispanic, and 8% of unspecified ethnicity. Additionally, the study’s racial composition was diverse, comprising 77% White, 17% African American, 1% American Indian or Alaska Native, 1% Asian, and 4% of unspecified race. We ensured that they were fully aware of the study’s purpose, procedures, and benefits, giving them the opportunity to ask questions and address any concerns beforehand. The study did not include any minors.

In this study, patients with lower extremity fractures underwent gait analysis using a validated chest-mounted IMU device [21]. Throughout the recovery process, they completed patient-reported outcome measures and participated in assessments focusing on 12 specific gait variables (Table 1). We preprocessed the raw data, analyzing variables such as movement efficiency, coordination, power generation, gait symmetry, stability, balance, temporal dynamics, and body orientation.

To evaluate the recovery rates from fractures in an uncomplicated context, we formed a cohort of 318 individuals who experienced no post-injury readmissions. Each patient had at least one follow-up visit within the first 200 weeks after their injury, allowing us to analyze which types of fractures demonstrate the highest rates of recovery.

The selection of patients without readmissions was intentional and important to the study’s design. This approach was adopted to mitigate any confounding factors that readmissions might introduce, whether directly related to the fractures or due to other medical complications. By focusing on this specific patient group, we can more accurately compare the recovery trajectories across all fracture types under observation. This method ensures that our findings reflect the natural progression of healing and rehabilitation, uncontaminated by external variables that could skew the data and confound the results.

Since we collected gait variable data for both the left and right sides, irrespective of the subjects’ injury side, throughout this study, we consider only the gait variables that correspond to the side of the injury. For example, if the injury side is left, we only consider the Mean Left Leg Lift Acceleration, which we then refer to simply as Mean Leg Lift Acceleration. Similarly, if the injury side is right, we only consider Mean Right Leg Lift Acceleration and again label it Mean Leg Lift Acceleration. This approach allows us to consistently combine data from both left and right injuries and to focus exclusively on the variables corresponding to the side of the injury, whether it is right or left.

Monitoring the gait analysis variables over time allows for the objective assessment of improvements and customization of rehabilitation programs. These variables act as precise markers of recovery, quantitatively enhancing evaluations alongside subjective assessments. However, the variability among individuals and the timing of the injury poses challenges in gait analysis. To overcome these, Linear Mixed Models (LMM) analysis [23] can be used to account for individual differences and the temporal effects on gait variables. The LMM model can be represented by the following formula:

(1) $$g_{ij}=\left(\beta_{0}+u_{0j}\right)+\left(\beta_{1}+u_{1j}\right)w_{ij}+\epsilon_{ij},$$

where, $g_{ij}$ represents the gait variable for patient i at the $j^{th}$ gait analysis visit, where j can take values of 1 or 2. Additionally, $w_{ij}$ represents the number of weeks that have passed since the injury for patient i at the $j^{th}\;$gait analysis visit. The model comprises both random effects and fixed effects [25], which enable a robust analysis. The random effects, represented by $u_{0j}$ and $u_{1j}$, capture the patient-specific deviations from the average intercept and slope, respectively. These random effects account for the inherent variability between patients and allow us to account for individual differences in the baseline gait variable value and the rate of change over time.

The fixed effects, represented by $\beta_{0}\;$and $\beta_{1}$, represent the average intercept and slope, respectively, across all patients. They provide insights into the overall relationships between the gait variable and the time elapsed since the injury. Moreover, $w_{ij}$ serves a dual purpose in this model. It acts as an input variable, capturing the effect of time since the injury on the gait variable g. Additionally, $w_{ij}$ is considered as a covariate [26,27] in the model. As a covariate, $w_{ij}$ addresses the specific challenge of uneven intervals between time points. By incorporating $w_{ij}$ as a covariate, we account for the uneven-varying nature of the gait analysis data and ensure the model appropriately adjusts for the effect of time elapsed since the injury on the gait variable g.

Finally, $\epsilon_{ij}$ represents the residual error or variation not explained by the fixed effects or random effects. It captures the inherent randomness in the gait variable measurements that cannot be accounted for by the model. Residuals are assumed to follow a normal distribution with mean zero and constant variance. Both random effects and residuals are assumed to be independent and identically distributed across patients and time points. When fitting the linear mixed model, the model estimates the fixed effects coefficients ($\beta_{0}\;$and $\beta_{1}$) and the variance components for the random effects ($u_{0j}$ and $u_{1j}$). The estimated coefficients provide quantitative information about the average effects of Weeks Since Injury on the gait variable g. The model’s output includes various statistical measures, such as *p*-values and confidence intervals, aiding in assessing the significance and precision of the estimated coefficients.

In this study, the choice to employ LMM rather than multivariate mixed-effects models was driven by several key considerations. First and foremost, the primary aim was to understand the relationship between the time elapsed since injury and specific gait variables individually rather than exploring multiple interdependent outcomes simultaneously. Furthermore, LMMs offer a more straightforward interpretation and easier management of data complexity when focusing on single outcomes. Multivariate models, which evaluate multiple dependent variables simultaneously, can yield results that are complex to interpret, especially when interactions between variables are considered. Given the specific objectives of our study, which require clear, actionable outcomes for clinical application, the simpler interpretative framework of LMMs was deemed more appropriate.

Additionally, the use of LMMs facilitates a robust handling of missing data and unbalanced time points across the dataset. In studies like ours, where follow-up times may vary and not all patients are assessed at every intended time point, LMMs efficiently handle this irregularity without compromising the statistical integrity of the results.

**Figure 1:**
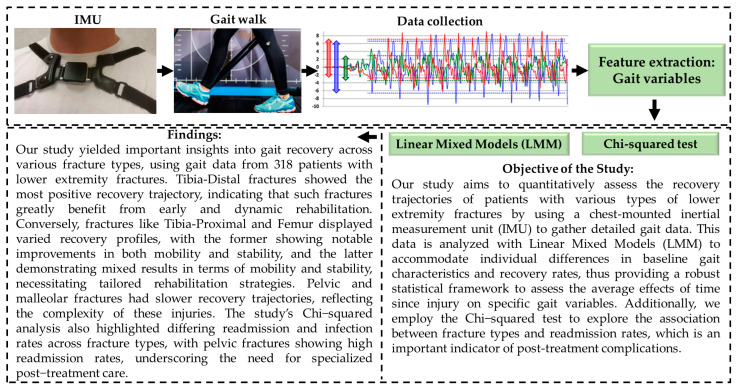
Schematic overview of the gait analysis methodology, objectives, and a summary of key findings from the study.

Following the analysis of patients without complications or readmissions, it is equally important to examine those who experienced readmissions during their recovery. This additional analysis allows us to develop a more comprehensive understanding of recovery trajectories and the challenges associated with different fracture types. To determine whether the type of fracture and the frequency of readmissions are statistically associated, we performed a Chi-squared test [24]. This test evaluates the relationship between fracture type and the number of readmissions, identifying whether specific fractures are more prone to complications necessitating readmissions. Figure 1 illustrates a schematic view of the gait analysis methodology, objectives, and a summary of our findings.

Among the various reasons for readmissions, infection stands out as a concern due to its significant impact on recovery outcomes and the potential for long-term complications. Understanding the prevalence of infection for each fracture type provides valuable insights into the risk profile associated with these injuries. By calculating the percentage of infections within the dataset for each fracture type, we aim to identify patterns that can inform clinical decision-making and targeted interventions.

## 3. Results

**Figure 2:**
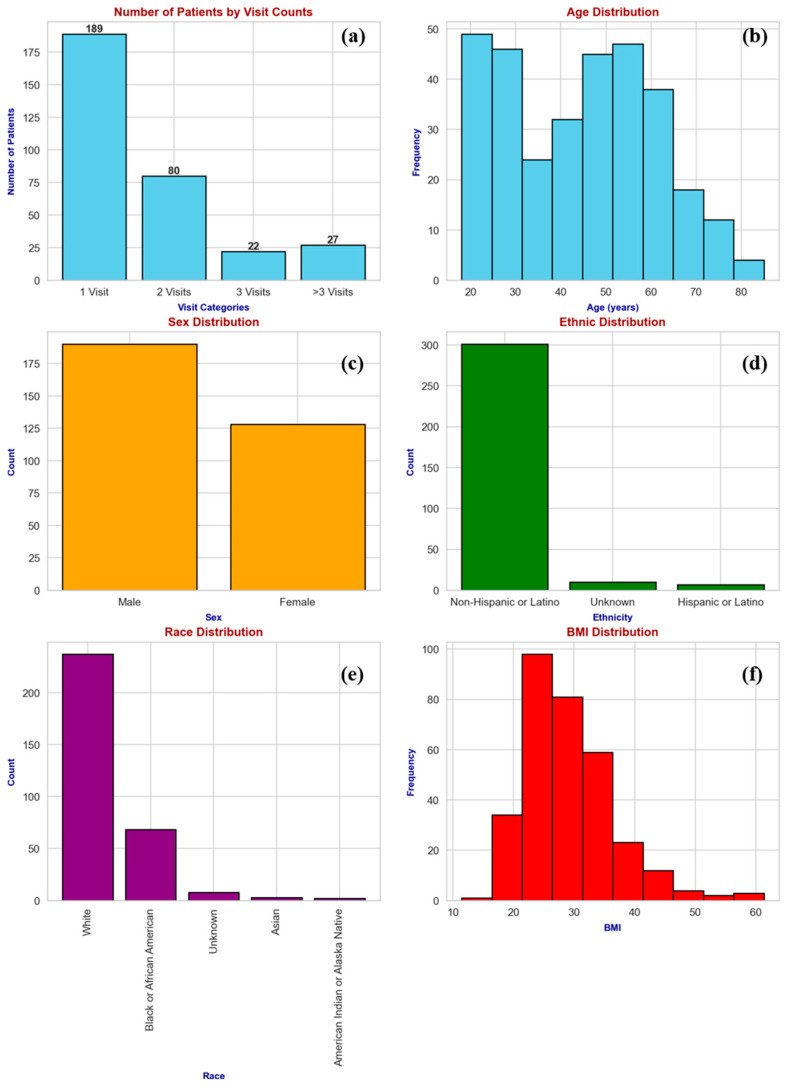
Distribution of visit frequencies and demographic characteristics of a 318-patient cohort recovering from fractures without readmissions or complications. This figure presents the number of follow-up visits each patient attended (**a**) and detailed demographic breakdowns, including age (**b**), sex (**c**), ethnicity (**d**), race (**e**), and BMI (**f**), illustrating the diversity and recovery monitoring within the cohort.

**Figure 3:**
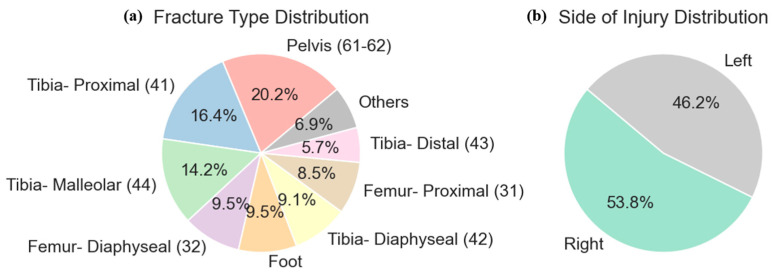
Distribution of fracture types and injury sides in the patient cohort without readmissions. (**a**) Pie chart showing the percentage distribution of different fracture types with a specific focus on the most common fractures and a collective category for less frequent types. (**b**) Pie chart depicting the distribution of injury sides, categorizing injuries as either left or right, which assists in identifying any asymmetry in injury occurrences.

Figure 2 illustrates the number of visits that each patient made and other demographic features such as age, sex, ethnicity, race, and BMI, which are important for understanding the diversity within the cohort and potential factors that could influence recovery patterns. We examined the patterns of fracture types and the distribution of injury sides within our patient cohort. Figure 3 illustrates these distributions, where Figure 3a presents the types of fractures observed, and Figure 3b details the side of the injury: left or right.

**Figure 4:**
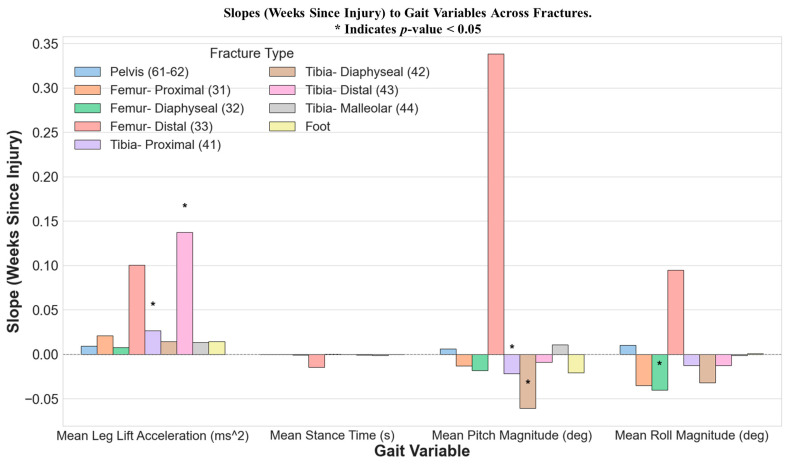
This bar graph displays the slopes from the LMM, illustrating the influence of weeks since injury on specific gait variables stratified by fracture type. Each bar denotes the magnitude and direction of the slope. Significant slopes (*p* < 0.05) are marked with an asterisk (*).

Figure 4 illustrates the coefficients (slope) of the LMM, highlighting the quantifiable impact of weeks since injury on specific gait variables across different types of lower extremity fractures. This figure captures the effects where the time since injury has shown a substantial influence on gait dynamics, as evidenced by the coefficients plotted for each variable.

To investigate whether the number of readmissions is associated with fracture type, we conducted a Chi-squared test. The results indicated a statistically significant relationship between fracture type and the number of readmissions (Chi-squared Test statistic: 35.684, *p*-value < 0.0001). This finding suggests that certain fracture types are more likely to result in readmissions compared to others.

**Figure 5:**
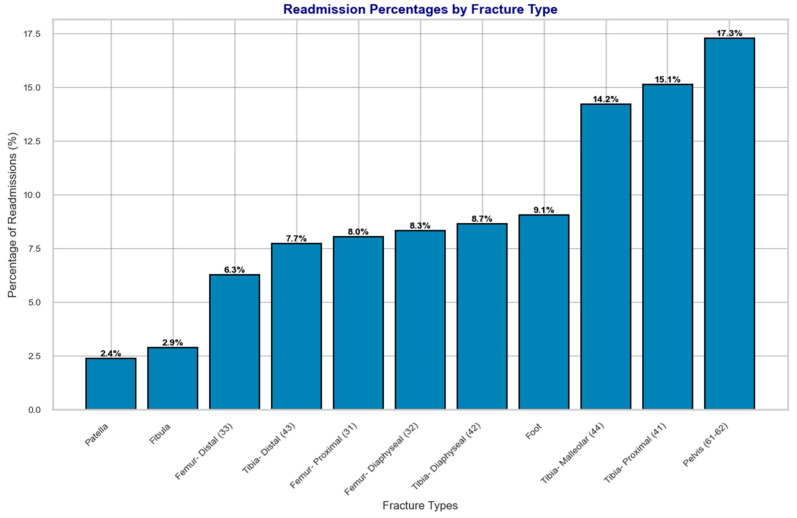
Distribution of patients with readmissions across different fracture types. The bar graph highlights the frequency of readmissions, showing variability in readmission rates among fracture types. This visualization underscores the significance of fracture-specific factors in influencing readmission rates.

**Figure 6:**
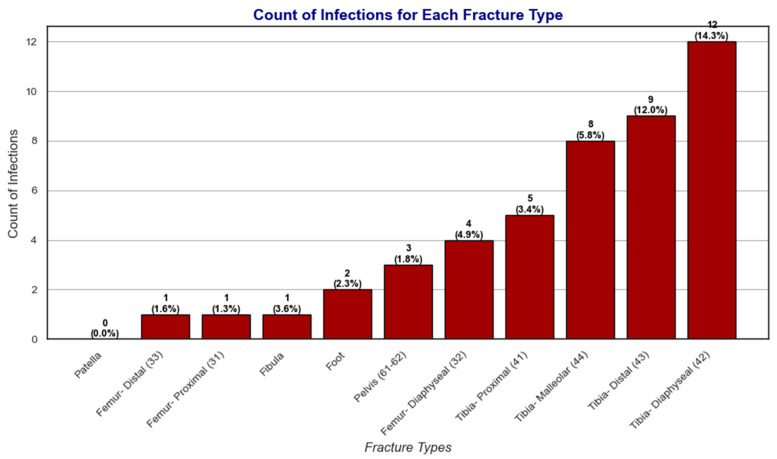
Distribution of patients with infection-related readmissions among fracture types. This figure focuses on the subset of patients whose readmissions were due to infection complications, illustrating how these events are distributed across various fracture types.

Figure 5 and Figure 6 provide a detailed visualization of these patterns. Figure 5 illustrates the distribution of patients with readmissions across different fracture types, highlighting variations in readmission frequency. Figure 6 focuses specifically on the subset of patients who experienced readmissions due to infection complications, offering insights into how infection-related readmissions are distributed among fracture types.

## 4. Discussion

The results of the LMM (see Figure 4) suggest distinct recovery trajectories for different fracture types, as reflected in the slopes of various gait variables over time. Among the fracture types, Tibia-Distal (43) demonstrated the fastest recovery progress, particularly in Mean Leg Lift Acceleration (${m/s}^{2}$), which had a significant positive slope (0.137225, *p* = 0.010797). This indicates rapid improvement in Leg Lift Acceleration, suggesting enhanced mobility and strength in the affected limb. Although the slopes for Tibia-Distal fractures, including Mean Stance Time (s), Mean Pitch Magnitude (deg), and Mean Roll Magnitude (deg), were not statistically significant, they still provide insights into the recovery dynamics. The slight negative slope in Mean Stance Time (s) (–0.000893) could indicate a gradual reduction in the time spent in the stance phase of the gait cycle, suggesting subtle improvements in gait efficiency. Similarly, the negative slope in Mean Pitch Magnitude (deg) (−0.009100) might reflect a trend toward reduced variability in sagittal plane motion, potentially indicative of greater stability and control as recovery progresses. Finally, the negative slope in Mean Roll Magnitude (deg) (−0.012644) suggests a decrease in side-to-side (coronal plane) motion, which could signify improved balance. Although these trends are not statistically significant, they are consistent with expected recovery patterns and underscore the gradual improvements across multiple dimensions of gait function for Tibia-Distal fractures.

Tibia-Proximal (41) also exhibited notable recovery progress, with significant improvement in Mean Leg Lift Acceleration (${m/s}^{2}$) (0.026469, *p* = 0.021521) and Mean Pitch Magnitude (deg) (−0.021914, *p* = 0.026378). The positive slope in Leg Lift Acceleration reflects better limb mobility and strength, while the negative slope in pitch magnitude suggests reduced sagittal plane variability, indicative of improved balance and stability. Although changes in Mean Stance Time (s) and Mean Roll Magnitude (deg) were not significant, the modest negative slope in roll magnitude (−0.012327) hints at subtle progress in gait efficiency and balance. These findings suggest that recovery in Tibia-Proximal fractures is characterized by simultaneous improvements in mobility and stability.

In contrast, Femur-Diaphyseal (32) displayed more limited recovery, with significant changes observed only in Mean Roll Magnitude (deg) (−0.040382, *p* = 0.011702). This reduction in roll magnitude likely reflects improved control in coronal plane motion and better balance during gait. The lack of significant changes in other gait variables, including Mean Leg Lift Acceleration (m/s^2^) (0.007733, *p* = 0.547054) and Mean Stance Time (s) (−0.000617, *p* = 0.169622), suggests that while balance improvements are evident, broader gains in mobility and gait efficiency may require more time or targeted intervention.

Femur-Distal (33) showed notable slopes in Mean Leg Lift Acceleration (m/s^2^) (0.100069), albeit without statistical significance. The high slope for Mean Leg Lift Acceleration suggests a substantial improvement in limb mobility and strength, potentially reflecting recovery efforts to regain dynamic movement. Femur-Proximal (31) demonstrated modest progress, as indicated by the positive slope in Mean Leg Lift Acceleration (m/s^2^) (0.020631). This suggests gradual improvement in lower-limb strength and mobility, although the slope is less pronounced than that of Distal femur fractures. The negative slopes in Mean Pitch Magnitude (deg) (−0.013163) and Mean Roll Magnitude (deg) (−0.035157) reflect a reduction in sagittal and coronal plane variability, respectively, suggesting progressive stabilization and control in gait mechanics.

Femur-Diaphyseal (32) showed the most pronounced changes in Mean Roll Magnitude (deg) (−0.040382), which was statistically significant (*p* = 0.011702). This indicates substantial improvements in lateral stability and control over time. However, the slope for Mean Leg Lift Acceleration (m/s^2^) (0.007733) was comparatively low, suggesting slower gains in mobility. The negative slope in Mean Pitch Magnitude (deg) (−0.018175) reflects a trend toward reduced sagittal plane variability, aligning with improved balance and gait regularity.

Pelvis (61–62) showed relatively small slopes across all gait variables, indicating gradual and less pronounced recovery progress. The positive slope in Mean Leg Lift Acceleration (m/s^2^) (0.008834) reflects slight improvements in limb strength and mobility. Tibia-Malleolar (44) displayed a modest positive slope in Mean Leg Lift Acceleration (m/s^2^) (0.013113), suggesting slight improvements in mobility. However, the slopes in Mean Pitch Magnitude (deg) (0.010781) and Mean Roll Magnitude (deg) (−0.001406) were small, indicating minimal variability changes in sagittal and coronal planes, respectively.

Tibia-Diaphyseal (42) demonstrated a small positive slope in Mean Leg Lift Acceleration (m/s^2^) (0.013983) and a significant reduction in Mean Pitch Magnitude (deg) (−0.060911, *p* = 0.012145). The former suggests gradual improvements in mobility, while the latter indicates enhanced sagittal plane control, reflecting better balance and coordination. The negative slope in Mean Roll Magnitude (deg) (−0.031750) highlights potential gains in lateral stability, although it was not statistically significant.

Note that within the 200-week follow-up period of our study, even the small slopes observed in various gait variables hold significant clinical relevance. These small but consistent changes in gait metrics, such as Mean Stance Time, Mean Pitch Magnitude, and Mean Roll Magnitude, are important indicators of gradual improvements in stability, balance, and efficiency of movement, which might not be immediately apparent but are essential for long-term recovery. While dramatic improvements might be expected shortly after medical interventions, these subtle shifts are indicative of the body’s ongoing adaptation and compensation mechanisms in response to the initial trauma and subsequent healing processes.

The Chi-squared test of independence ($\chi^{2}$) revealed a statistically significant association between fracture type and the frequency of readmissions ($\chi^{2}=35.68,p=10\times 10^{-5}$). This finding underscores the varying risk profiles of different fractures and their susceptibility to complications requiring hospital readmissions. By analyzing the percentages of readmissions for each fracture type (Figure 5), along with the infection rates (Figure 6), we gain valuable insights into the challenges associated with recovery from these injuries.

Fractures of the pelvis (61–62) exhibited the highest rate of readmissions at 17.3%, marking them as particularly prone to complications. Despite this, the infection rate for pelvic fractures was relatively low (1.8%), suggesting that other factors, such as pain management, immobility, or comorbid conditions, may play a significant role in driving readmissions for this fracture type.

Similarly, Tibia-Proximal (41) fractures had the second-highest readmission rate at 15.1%, accompanied by a moderate infection rate of 3.4%. This suggests a dual burden of complications, potentially stemming from the complexity of Proximal tibia fractures and their associated surgical interventions.

The Tibia-Malleolar (44) fractures presented a distinct profile, with a high readmission rate of 14.2% and an infection rate of 5.8%. This indicates that infections may significantly contribute to readmissions in this fracture type, potentially due to their proximity to the skin and soft tissue, making them more susceptible to wound-related complications.

On the other hand, fractures such as Tibia-Distal (43) and Tibia-Diaphyseal (42) also stood out with readmission rates of 7.7% and 8.6%, respectively, coupled with notably high infection rates (12.0% and 14.3%). These findings suggest that Distal and Diaphyseal tibia fractures face elevated risks of infection, potentially due to factors like compromised blood supply, open fractures, or prolonged immobilization.

Femur fractures, including Proximal (31), Distal (33), and Diaphyseal (32), exhibited intermediate rates of readmissions, ranging from 6.3% for Distal femur fractures to 8.3% for Diaphyseal femur fractures. Interestingly, Distal femur fractures had one of the lowest infection rates (1.6%), suggesting that non-infectious complications such as joint stiffness, delayed union, or hardware failure may contribute to readmissions. Conversely, Diaphyseal femur fractures had a higher infection rate of 4.9%, indicating a mixed profile of risks, including infections and potential mechanical issues.

Proximal femur fractures, while displaying a moderate readmission rate of 8.0%, had a relatively low infection rate of 1.3%, highlighting the importance of addressing other factors, such as rehabilitation challenges or age-related vulnerabilities, that may complicate recovery in this fracture type. Fractures of the foot had a readmission rate of 9.1%, with an infection rate of 2.3%. While these rates are moderate, they likely reflect the complexity of managing weight-bearing fractures and the challenges of returning to ambulation.

Similarly, fractures of the fibula exhibited a lower readmission rate (2.9%) but a relatively high infection rate (3.6%). This suggests that while fibula fractures are less likely to lead to readmissions overall, infections can be a significant concern in cases requiring surgical intervention. Patella fractures, with the lowest readmission rate (2.4%) and no recorded infections, appear to have the least complicated recovery profile among the fracture types analyzed.

In summary, the Chi-squared analysis and subsequent infection and readmission rate data reveal distinct risk profiles for different fracture types. Pelvis, Tibia-Proximal, and Tibia-Malleolar fractures emerge as the most problematic in terms of overall readmissions, while Tibia-Distal and Tibia-Diaphyseal fractures show high infection rates, highlighting the need for targeted infection prevention strategies. Femur fractures and foot fractures display moderate complication rates, while patella fractures exhibit a relatively low risk of complications. These findings emphasize the importance of tailoring post-fracture management strategies to the unique challenges posed by each fracture type, with a focus on mitigating infection risks and addressing factors contributing to readmissions. Future studies could further explore the specific causes of readmissions and the impact of individualized interventions on improving recovery outcomes.

## 5. Conclusions

Our study offered important insights into gait recovery across different types of lower extremity fractures, highlighting the significance of early gait dynamics and the possibility of personalized rehabilitation strategies. By analyzing gait data from 318 patients who underwent successful recovery without complications or readmissions, we used a chest-mounted inertial measurement unit (IMU) to capture detailed gait metrics such as Mean Leg Lift Acceleration, stance time, pitch, and roll magnitudes. The use of linear mixed model (LMM) analysis provided an objective assessment of gait variables over time, allowing us to understand individual recovery dynamics and average trajectories across fracture types. Among these, Tibia-Distal fractures showed the most positive recovery trajectory, marked by rapid improvements in mobility and strength, suggesting effective responses to dynamic movement and early mobilization. Moreover, trends of reduced variability in pitch and roll magnitudes indicated improving gait efficiency and stabilization over time.

The study also found notable recovery progress in Tibia-Proximal fractures, with simultaneous improvements in mobility and stability, suggesting benefits from rehabilitation that focuses on strength and balance. Femur fractures exhibited varied recovery patterns; Diaphyseal femur fractures improved in stability but showed slower mobility progress, while Distal femur fractures had mixed results in strength and stability, underlining the individualized nature of recovery for these injuries. Pelvic and malleolar fractures demonstrated slower recovery trajectories due to challenges in core stability and maintaining joint integrity, respectively. Chi-squared analysis revealed variability in readmission and infection rates across fracture types, with pelvic fractures showing the highest readmission rates and a need for enhanced infection control in fractures like Distal and Diaphyseal tibia.

### Limitations

This study has limitations to consider. The follow-up period, while extensive, may not fully capture long-term recovery patterns that occur after the study ends, potentially missing later complications and recovery trajectories. Additionally, the use of a chest-mounted IMU for gait analysis could introduce biases due to device positioning and technology limitations like signal drift. While measurements were validated against a gold-standard system, differences in technology could still affect the results. Furthermore, the study focuses on general gait variables without evaluating specific gait characteristics like body movement angles or step asymmetry, which could provide deeper insights into biomechanical changes during recovery.

## Figures and Tables

**Table 1:** Summary of gait variables and descriptions.

Variable	Unit	Description
Mean Left Leg Lift Acceleration	m/s^2^	The average acceleration of the left leg during the lifting phase of the gait cycle.
Left Leg Lift Acceleration SEM	m/s^2^	Standard Error Mean (SEM) of the acceleration of the left leg during the lifting phase of the gait cycle.
Mean Right Leg Lift Acceleration	m/s^2^	The average acceleration of the right leg during the lifting phase of the gait cycle.
Right Leg Lift Acceleration SEM	m/s^2^	Standard Error Mean (SEM) of the acceleration of the right leg during the lifting phase of the gait cycle.
Mean Left Stance Time	s	The average duration of the left leg’s stance phase, which is the period when the foot is in contact with the ground.
Left Stance Time SEM	s	Standard Error Mean (SEM) of the duration of the left leg’s stance phase.
Mean Right Stance Time	s	The average duration of the right leg’s stance phase, which is the period when the foot is in contact with the ground.
Right Stance Time SEM	s	Standard Error Mean (SEM) of the duration of the right leg’s stance phase.
Mean Pitch Magnitude	degrees°	The average magnitude of the pitch (forward–backward) movement of the body during walking.
Pitch Magnitude SEM	degrees°	Standard Error Mean (SEM) of the magnitude of the pitch (forward–backward) movement of the body during walking.
Mean Roll Magnitude	degrees°	The average magnitude of the roll (side-to-side) movement of the body during walking.
Roll Magnitude SEM	degrees°	Standard Error Mean (SEM) of the magnitude of the roll (side-to-side) movement of the body during walking.

## Data Availability

The data that support the findings of this study are not publicly available due to privacy and ethical restrictions, as they contain information that could compromise the privacy of research participants. The data involve potentially identifiable patient information, and sharing these data would violate the confidentiality agreements under which the data were collected.

## References

[1] Hoyt B.W., Pavey G.J., Pasquina P.F., Potter B.K. (2015). Rehabilitation of lower extremity trauma: A review of principles and military perspective on future directions. Curr. Trauma Rep..

[2] Haller Justin M., Potter Michael Q., Kubiak Erik N. (2013). Weight bearing after a periarticular fracture: What is the evidence?. Orthop. Clin..

[3] Hyer C.F., Atway S., Berlet G.C., Lee T.H. (2010). Early weight bearing of calcaneal fractures fixated with locked plates: A radiographic review. Foot Ankle Spec..

[4] Buckley R., Tough S., McCormack R., Pate G., Leighton R., Petrie D., Galpin R. (2002). Operative compared with nonoperative treatment of displaced intra-articular calcaneal fractures: A prospective, randomized, controlled multicenter trial. J. Bone Jt. Surg..

[5] Bacon S., Smith W.R., Morgan S.J., Hasenboehler E., Philips G., Williams A., Ziran B.H., Stahel P.F. (2008). A retrospective analysis of comminuted intra-articular fractures of the tibial plafond: Open reduction and internal fixation versus external Ilizarov fixation. Injury.

[6] Zarek S., Othman M., Macias J. (2002). The Ilizarov method in the treatment of pilon fractures. Ortop. Traumatol. Rehabil..

[7] Joslin C., Eastaugh-Waring S., Hardy J., Cunningham J. (2008). Weight bearing after tibial fracture as a guide to healing. Clin. Biomech..

[8] Kershaw C.J., Cunningham J.L., Kenwright J. (1993). Tibial external fixation, weight bearing, and fracture movement. Clin. Orthop. Relat. Res..

[9] Arazi M., Ögün T.C., Oktar M.N., Memik R., Kutlu A. (2001). Early weight-bearing after statically locked reamed intramedullary nailing of comminuted femoral fractures: Is it a safe procedure?. J. Trauma Acute Care Surg..

[10] Brumback R.J., Toal T.R., Murphy-Zane M.S., Novak V.P., Belkoff S.M. (1999). Immediate weight-bearing after treatment of a comminuted fracture of the femoral shaft with a statically locked intramedullary nail. J. Bone Jt. Surg. Am..

[11] Herrera A., Domingo J., Martinez A. (2008). Results of osteosynthesis with the ITST nail in fractures of the trochanteric region of the femur. Int. Orthop..

[12] Kazemi N., Archdeacon M.T.M. (2012). Immediate full weightbearing after percutaneous fixation of anterior column acetabulum fractures. J. Orthop. Trauma.

[13] Conn K.S., Parker M.J. (2004). Undisplaced intracapsular hip fractures: Results of internal fixation in 375 patients. Clin. Orthop. Relat. Res..

[14] Koval K.J., Sala D.A., Kummer F.J., Zuckerman J.D. (1998). Postoperative weight-bearing after a fracture of the femoral neck or an intertrochanteric fracture. J. Bone Jt. Surg. Am..

[15] Whittle M.W. (2014). Gait Analysis: An Introduction.

[16] Hofman M., Kolejewska A., Greven J., Andruszkow H., Kobbe P., Tolba R., Hildebrand F., Poeze M. (2020). Gait analysis and muscle weight analysis after lower extremity fractures in a small animal model. Gait Posture.

[17] Cappozzo A., Della Croce U., Leardini A., Chiari L. (2005). Human movement analysis using stereophotogrammetry: Part 1: Theoretical background. Gait Posture.

[18] Chiari L., Della Croce U., Leardini A., Cappozzo A. (2005). Human movement analysis using stereophotogrammetry: Part 2: Instrumental errors. Gait Posture.

[19] Warschawski Y., Elbaz A., Segal G., Norman D., Haim A., Jacov E., Grundshtein A., Steinberg E. (2015). Gait characteristics and quality of life perception of patients following tibial plateau fracture. Arch. Orthop. Trauma Surg..

[20] Millar S.C., Bennett K., Fraysse F., Arnold J.B., Solomon L.B., Thewlis D. (2020). Longitudinal changes in lower limb joint loading up to two years following tibial plateau fracture. Gait Posture.

[21] Swart E., Peindl R., Zheng N., Habet N., Churchill C., Ruder J.A., Seymour R., Karunakar M., Kellam J., Sims S. (2019). Electronically augmented gait abnormality assessment following lower extremity trauma. OTA Int..

[22] Rezapour M., Seymour R.B., Sims S.H., Karunakar M.A., Habet N., Gurcan M.N. (2024). Employing machine learning to enhance fracture recovery insights through gait analysis. J. Orthop. Res..

[23] Jiang J., Nguyen T. (2007). Linear and Generalized Linear Mixed Models and Their Applications.

[24] Greenwood P.E., Nikulin M.S. (1996). A Guide to Chi-Squared Testing.

[25] Firebaugh G., Warner C., Massoglia M. (2013). Fixed effects, random effects, and hybrid models for causal analysis. Handbook of Causal Analysis for Social Research.

[26] Bates D. (2005). Fitting linear mixed models in R. R News.

[27] Qian T., Klasnja P., Murphy S.A. (2020). Linear mixed models with endogenous covariates: Modeling sequential treatment effects with application to a mobile health study. Stat. Sci. Rev. J. Inst. Math. Stat..

